# Exercise alters the molecular pathways of insulin signaling and lipid handling in maternal tissues of obese pregnant mice

**DOI:** 10.14814/phy2.14202

**Published:** 2019-08-29

**Authors:** Barbara Musial, Denise S. Fernandez‐Twinn, Daniella Duque‐Guimaraes, Sarah K. Carr, Abigail L. Fowden, Susan E. Ozanne, Amanda N. Sferruzzi‐Perri

**Affiliations:** ^1^ Centre for Trophoblast Research, Department of Physiology, Development and Neuroscience University of Cambridge Cambridge United Kingdom; ^2^ MRC Metabolic Disease Unit University of Cambridge Metabolic Research Laboratories, Wellcome Trust‐MRC Institute of Metabolic Science, Addenbrooke’s Hospital Cambridge United Kingdom

**Keywords:** Exercise, insulin resistance, lipid metabolism, obesity, pregnancy

## Abstract

Obesity during gestation adversely affects maternal and infant health both during pregnancy and for long afterwards. However, recent work suggests that a period of maternal exercise during pregnancy can improve metabolic health of the obese mother and her offspring. This study aimed to identify the physiological and molecular impact of exercise on the obese mother during pregnancy that may lead to improved metabolic outcomes. To achieve this, a 20‐min treadmill exercise intervention was performed 5 days a week in diet‐induced obese female mice from 1 week before and up to day 17 of pregnancy. Biometric, biochemical and molecular analyses of maternal tissues and/or plasma were performed on day 19 of pregnancy. We found exercise prevented some of the adverse changes in insulin signaling and lipid metabolic pathways seen in the liver, skeletal muscle and white adipose tissue of sedentary‐obese pregnant dams (p110*β*, p110*α*, AKT, SREBP). Exercise also induced changes in the insulin and lipid signaling pathways in obese dams that were different from those observed in control and sedentary‐obese dams. The changes induced by obesity and exercise were tissue‐specific and related to alterations in tissue lipid, protein and glycogen content and plasma insulin, leptin and triglyceride concentrations. We conclude that the beneficial effects of exercise on metabolic outcomes in obese mothers may be related to specific molecular signatures in metabolically active maternal tissues during pregnancy. These findings highlight potential metabolic targets for therapeutic intervention and the importance of lifestyle in reducing the burden of the current obesity epidemic on healthcare systems.

## Introduction

Worldwide, the prevalence of obesity is increasing rapidly (Collaboration, N.R.F. 2016). In 2014, the World Health Organisation reported that >1.9 billion adults (39% of the population) were overweight and >600 million (13%) were obese (WHO 2016). In the UK, recent findings have indicated that >50 % of women of reproductive age and >30% of pregnant women are overweight or obese (Davies 2015). This is particularly concerning, as obesity during pregnancy has detrimental effects on the mother and infant both during pregnancy and in later life. In obese women and experimental animals, the metabolic responses to pregnancy are often exaggerated, with a greater degree of hyperglycemia and hyperinsulinemia in late gestation compared to leaner mothers (Lacroix et al. 2013; Carter et al. 2015; Rosario et al. 2015; Fernandez‐Twinn et al. 2017; Musial et al. 2017). There is also evidence for defects in the abundance of insulin signaling and lipid metabolic proteins in maternal skeletal muscle, white adipose tissue and/or liver that may relate to altered glucose‐insulin handling and nutrient partitioning in human and rodent pregnancies associated with obesity (Barbour et al. 2007; Sferruzzi‐Perri et al. 2013b; Fernandez‐Twinn et al. 2017; Musial et al. 2017). However, detailed information of the molecular changes occurring in metabolic organs of obese mothers in any mammalian species during pregnancy is incompletely understood. This is important as women who are obese are at increased risk of pregnancy complications including gestational diabetes mellitus (GDM) and preeclampsia and are more likely to develop obesity, type‐2 diabetes and cardiovascular disease later in life (Frias and Grove 2012; Marchi et al. 2015; Berggren et al. 2016). Their infants are also more prone to intrauterine growth and developmental abnormalities, perinatal complications and to adult obesity, type‐2 diabetes and heart disease themselves (Frias and Grove 2012; Marchi et al. 2015). Obesity during pregnancy therefore, creates a vicious cycle of in utero transmission of cardiometabolic disease from mother to child beyond genetic inheritance (Catalano 2003). Thus, interventions to improve pregnancy outcome and to halt this cycle of intergenerational disease transmission would have a major impact on population health.

Exercise or physical activity is advocated as the nonpharmacological intervention to combat the metabolic dysfunction of obesity (Knowler et al. 2002; Organisation 2010). The UK Medical Health Office also recently endorsed physical activity for women during pregnancy (Recommendations, U.C.M.O. 2017). Exercise is known to improve whole body glucose tolerance, lipid handling and insulin sensitivity and thereby, reduces the risk of type‐2 diabetes and metabolic syndrome in nonpregnant humans (Knowler et al. 2002; Conn et al. 2014) and rodents (Bradley et al. 2008; Aoi et al. 2011; Krawczewski Carhuatanta et al. 2011; Higa et al. 2014; Jordy et al. 2015; Tsuzuki et al. 2015; Stanford et al. 2015b; Ko et al. 2018; Rattanavichit et al. 2018). Exercise upregulates the expression and activity of insulin signaling components and increases glucose uptake in skeletal muscle in both lean and obese nonpregnant animals (Kim et al. 1999; Chibalin et al. 2000; Hawley and Lessard 2008). It also promotes fatty acid oxidation and reduces lipid synthesis in the liver, skeletal muscle, and white adipose tissue in lean rats (Ruderman et al. 2003). Exercise also has positive effects during pregnancy, with beneficial outcomes for both mother and her child. Exercise prior to and/or during pregnancy can prevent excessive gestational weight gain (Mottola et al. 2010; Muktabhant et al. 2015), the development of GDM (Russo et al. 2015; Wang et al. 2017) and the need for insulin use in women with GDM (Brankston et al. 2004; de Barros et al. 2010). Similarly, exercise improved insulin sensitivity, lipid metabolism, and glucose tolerance in diabetic and obese rodent pregnancies (Huang et al. 2018) and glucose tolerance, insulin sensitivity, and dyslipidemia during obese rodent pregnancy (Carter et al. 2015; Vega et al. 2015; Fernandez‐Twinn et al. 2017). In mice, exercise also ameliorates the increased levels of maternal oxidative stress (Vega et al. 2015) and placental hypoxia and lipid accumulation associated with maternal obesity (Fernandez‐Twinn et al. 2017). Furthermore, there is accumulating evidence that maternal exercise has beneficial effects on cardiometabolic outcomes of the offspring in overweight or obese women (Barakat et al. 2016; Patel et al. 2017) and in experimental animals (Carter et al. 2012; Carter et al. 2013; Rajia et al. 2013; Laker et al. 2014; Blaize et al. 2015; Raipuria et al. 2015; Vega et al. 2015; Stanford et al. 2015a; Fernandez‐Twinn et al. 2017; Quiclet et al. 2017; Ribeiro et al. 2017; Stanford et al. 2017; Beeson et al. 2018; Cunningham et al. 2018). However, the physiological and molecular impact of exercise on the metabolically active tissues of the mother, which may lead to improved pregnancy outcomes, remain unknown. Thus, the aim of this study was, using a mouse model, to determine the effects of obesity during pregnancy with and without an exercise intervention on maternal body weight and composition, nutrient handling and insulin and lipid signaling in liver, skeletal muscle, and adipose tissue. These findings were then related to maternal glucose tolerance and the growth of the conceptus.

## Materials and Methods

### Animals

All experiments were carried out in accordance with the UK Home Office Animals (Scientific Procedures) Act 1986 following ethical review by the University of Cambridge Animal Welfare and Ethical Review Board. The dietary method of inducing obesity and the exercise intervention have been described previously (Fernandez‐Twinn et al. 2017). Briefly, C57Bl/6 females (6 weeks of age, *n* = 20) that were purchased from Charles River, UK were group housed under dark: light 12:12 conditions and were randomly assigned to be fed for 6 weeks before their first pregnancy and thereafter one of two diets ad libitum; either a standard RM1 diet (7% simple sugars, 3% fat, 50% polysaccharide, 15% protein [w/w], 10.74 kJ/g) or a semi‐synthetic energy‐rich highly palatable obesogenic diet (10% simple sugars, 20% animal lard, 28% polysaccharide, 23% protein [w/w], 28.43 kJ/g) supplemented with sweetened condensed milk (Nestle, UK) (16% fat, 33% simple sugars, 15% protein, 13.7 kJ/g), which was supplemented with the vitamin and mineral mix AIN93G. Both diets were purchased from Special Dietary Services (Witham, UK diet). Dams were allowed to litter their first pregnancy and their pups were culled at weaning. The first pregnancy ensured fertility and nurturing in the experimental mice. Six control dams and fourteen obese dams were then mated with control males fed the standard RM1 chow to generate a second pregnancy. The dams were maintained on their respective diets throughout their second pregnancy (copulatory plug defined as day 1 of pregnancy). It is known that this diet is associated with hyperinsulinemia and glucose intolerance in the dam and this model of preexisting obesity leads to small for gestational age pups (Fernandez‐Twinn et al. 2017). Five of the obese dams were exercised for 20 min during their dark/active phase by treadmill running, at least one week before their second pregnancy (5 m/min with daily incremental increases in speed to 12.5 m/min by day 5) and then until day 17 of pregnancy (12.5 m/min per day, only on weekdays). We specifically chose to commence the exercise regime prior to pregnancy as this would model those women who are obese and trying to improve their health status prior to pregnancy. The intensity and duration of daily exercise was also chosen to be moderate (approximates to 5–10% of the distance that a nonpregnant female mouse would run voluntarily, if given a wheel (Koteja et al. 1999; Smythe and White, 2011)) and would be a feasible exercise level for obese women. This degree of activity was achievable by all mice without any enforcement.

At the start of the study, the number of animals per group was 6, 9 and 5 for control, obese sedentary and obese exercised. However, two obese sedentary dams were not included in the final analysis as these mice did not gain the threshold of 10 g body fat prior to mating, which was required for inclusion in the obese group. Thus, the final number of animals per group was 6, 7, and 5 for control, obese sedentary, and obese exercised.

### Tissue and blood collection

On day 19 of pregnancy, dams were killed by CO_2_ asphyxiation. Maternal liver, skeletal muscle (*Biceps femoris)*, white adipose tissue (WAT, retroperitoneal fat), kidneys, heart, placentas, and fetuses were weighed (excl. skeletal muscle) and immediately snap frozen on dry ice for biochemical analysis and quantification of protein abundance. Blood was collected for plasma analyses. All tissues and plasma samples were collected from our dams under fed conditions to avoid introducing stress as a confounder.

### Serum analyses

Leptin and insulin were measured by enzyme‐linked immunosorbent assay, ELISA (Ultra Sensitive Mouse Insulin ELISA Kit and Mouse Leptin ELISA Kit, both from Crystal Chem, Zaandam, Netherlands). Samples were assayed in duplicate and intra and inter‐assay coefficients of variation of less than 10.0% was considered acceptable for both assays. Serum triglycerides, nonesterified fatty acids, and total cholesterol concentrations were measured by the Medical Research Council Metabolic Diseases Unit Mouse Biochemistry Laboratory (Addenbrooke's Hospital, UK).

### Tissue biochemical analyses

Glycogen, fat, and protein content were quantified using standard biochemical assays (Musial et al. 2016, 2017). The abundance of proteins in the insulin signaling and lipid metabolic pathway was determined by Western blotting with commercially available antibodies (Table 1) as described previously (Musial et al. 2016, 2017). In particular, we loaded 75 *µ*g of protein per sample and measured the receptors that can respond to insulin (insulin receptor, InsR and type 1 insulin‐like growth factor receptor, IGF1R), mitogen‐activated protein kinase (MAPK), which regulates cell proliferation and phosphoinositide‐3 kinases (PI3K p85*α* regulatory subunit, p110*α* and p110*β* catalytic subunits) and protein kinase B (AKT) that transduce the metabolic actions of insulin. We assessed the abundance of a glucose storage enzyme (glycogen synthase kinase‐3, GSK3), proteins that control protein translation downstream of the mechanistic target of rapamycin, mTORC1 (ribosomal S6 kinase, S6K and eukaryotic translation initiation factor 4E‐binding protein, 4EBP) and lipid metabolic proteins (sterol regulatory element‐binding protein, SREBP; peroxisome proliferator‐activated receptors, PPARs; lipoprotein lipase, LPL; fatty acid transport protein, FATP1, and fatty acid synthase, FAS). The ponceau‐stained membranes confirmed equal protein loading and transfer.

**Table 1:** The details of the antibodies used for detection of protein abundance by western blotting.

Primary antibody	Species	Manufacturer	Catalog no.	Dilution
InsR	Rabbit	Santa Cruz	sc‐711	1/400
IGF1R	Rabbit	Santa Cruz	sc‐713	1/400
p110*α*	Rabbit	Cell signaling	4249	1/1000
p110*β*	Rabbit	Cell signaling	3011	1/1000
p85*α*	Rabbit	Millipore	06–195	1/5000 in 1% milk
Akt	Rabbit	Cell signaling	9272	1/1000
pAkt Thr308	Rabbit	Cell signaling	9275	1/1000
pAkt Ser473	Rabbit	Cell signaling	9271	1/1000
GSK3	Rabbit	Cell signaling	9315	1/1000
pGSK3 Ser21/9	Rabbit	Cell signaling	9331	1/1000
S6K	Rabbit	Cell signaling	2708	1/1000
pS6K Thr389	Rabbit	Cell signaling	9234	1/1000
4EBP	Rabbit	Cell signaling	9644	1/1000
p4EBP Ser65	Rabbit	Cell signaling	9451	1/1000
MAPK	Rabbit	Cell signaling	4695	1/1000
pMAPK Thr202/Tyr204	Rabbit	Cell signaling	4370	1/1000
PEPCK	Rabbit	Santa Cruz	sc‐32789	1/200
G6Pase	Goat	Santa Cruz	sc‐27198	1/200
LPL	Mouse	Abcam	21356	1/1000
SREBP	Mouse	Abcam	3259	1/200
FAS	Rabbit	Cell signaling	3180	1/1000
PPAR*α*	Rabbit	Abcam	8934	1/1500
PPAR*γ*	Mouse	Santa Cruz	sc‐7273	1/200
FATP1	Goat	Santa Cruz	sc‐31955	1/400

### Statistics

To compare all three groups of mice, data were analysed by one‐way analysis of variance (ANOVA), followed by pairwise Bonferroni post hoc analyses (significant differences were denoted by different superscripts). Relationships between variables were assessed by Pearson (*r*) correlation. All statistical analyses were performed on GraphPad Prism 4.0 and findings *P* < 0.05 were considered significant.

## Results

### Maternal biometry, biochemical composition, and concentrations of metabolites and hormones

We have previously shown that obese sedentary and exercised mice have increased adiposity and reduced lean body mass compared to control dams (Fernandez‐Twinn et al. 2017). Furthermore, maternal adipose and lean mass was not affected by exercise in obese dams (Fernandez‐Twinn et al. 2017). Consistent with these findings, hysterectomized weights of the obese sedentary and obese exercised dams were similar and greater than that of control dams (Table 2). The weight of the maternal WAT depot was greater in sedentary obese dams compared to control (Table 2). The weight of the maternal heart and kidneys were not different between control and sedentary obese dams. Litter size was unaffected but total conceptus mass was reduced by maternal obesity regardless of whether dams were sedentary or exercised (Table 2). The reduction in conceptus mass was related to a decrease in fetal, but not placental weight in obese sedentary and obese exercised dams (Table 2).

**Table 2:** The effects of obesity with and without exercise intervention on maternal biometry during pregnancy.

Weights	Control	Obese	Exercised
Hysterectomized weight	28 ± 0.5^a^	38 ± 1.9^b^	35 ± 2.0^b^
Liver (mg)	1949 ± 99^a^	2213 ± 100^ab^	2399 ± 154^b^
Kidney (mg)	352 ± 18	407 ± 20	414 ± 36
Heart (mg)	163 ± 19	161 ± 6	179 ± 16
Retroperitoneal fat (mg)	173 ± 21^a^	964 ± 152^b^	876 ± 159^b^
Total conceptus weight* (mg)	12405 ± 382^a^	9454 ± 555^b^	10164 ± 629^b^
Total placental weight (mg)	854 ± 50	746 ± 47	869 ± 45
Total fetal weight (mg)	11551 ± 389^a^	8708 ± 532^b^	9296 ± 661^b^
Litter size	10 ± 0.3	9 ± 0.6	9.4 ± 0.6

Data are expressed as mean ± SEM (control *n* = 6, obese *n* = 6–7, exercised *n* = 5 dams/litters). Values with different superscripts are significantly different from each other (*P* < 0.05 one way ANOVA, post hoc Bonferroni test).

* Total conceptus mass is the sum of all fetuses and placentas in the litter.

Exercise did not affect the biochemical composition of the liver, skeletal muscle, or WAT of obese dams (Table 3). The protein content of the liver was reduced and its fat content increased to a similar extent in both groups of obese dams relative to the control group (Table 3). Fat content in the skeletal muscle and WAT was also elevated and protein content of the WAT decreased in obese dams irrespective of whether they were exercised or not (Table 3).

**Table 3:** The effects of obesity with and without exercise intervention on maternal tissue composition during pregnancy.

Tissue content	Control	Obese	Exercised
Liver			
Protein content (mg/g)	172 ± 1.8^a^	147 ± 3.8^b^	149 ± 10.7^b^
Glycogen (mg/g)	39 ± 4.9^a^	27 ± 2.1^ab^	23 ± 4.0^b^
Total glycogen (mg)	77 ± 11.6	61 ± 4.3	55 ± 9.1
Fat content (%)	5.2 ± 0.2^a^	18.8 ± 2.0^b^	15.3 ± 0.9^b^
Total fat content (mg)	101 ± 7.3^a^	414 ± 42^b^	369 ± 35^b^
Skeletal muscle			
Protein content (mg/g)	59 ± 3.4	62 ± 2.4	58 ± 2.0
Fat content (%)	6.6 ± 1.5^a^	20.8 ± 3.3^b^	27.7 ± 4.6^b^
White adipose tissue			
Protein content (mg/g)	35 ± 3.1^a^	16 ± 2.6^b^	14 ± 3^b^

Data are expressed as mean ± SEM (control *n* = 6, obese *n* = 6–7, exercised *n* = 5 dams). Values with different superscripts are significantly different from each other (*P* < 0.05 one way ANOVA, post hoc Bonferroni test).

Obese dams had a lower concentration of circulating triglycerides compared to controls (Table 4). However, the concentrations of glucose, cholesterol and nonesterified fatty acids in the maternal circulation were not affected by obesity or exercising obese dams (Table 4). Insulin and leptin concentrations for lean and sedentary and exercised obese animals have already been published (Fernandez‐Twinn et al. 2017) but also shown in Table 3 for clarity. These data show that maternal plasma leptin and insulin were increased by obesity and insulin partially normalized to control levels in response to exercise in obese dams ((Fernandez‐Twinn et al. 2017) and Table 4).

**Table 4:** The effects of obesity with and without exercise intervention on maternal metabolites and hormones during pregnancy.

Metabolite/hormone	Control	Obese	Exercised
Fed glucose (mmol/L)	9.2 ± 1.0	9.5 ± 0.7	10.6 ± 0.8
Nonesterified fatty acids (*μ*mol/L)	831 ± 266	713 ± 125	455 ± 31
Triglycerides (mmol/L)	1.1 ± 0.1^a^	0.7 ± 0.05^b^	0.7 ± 0.06^b^
Cholesterol (mmol/L)	1.3 ± 0.1	1.5 ± 0.1	1.3 ± 0.1
Insulin (pmol/L)	119 ± 29^a^	282 ± 49^b^	180 ± 34^ab^
Leptin (pmol/L)	136 ± 12^a^	486 ± 87^b^	346 ± 19^b^

Data are expressed as mean ± SEM (control *n* = 6, obese *n* = 6–7, exercised *n* = 5 dams). Values with different superscripts are significantly different from each other (*P* < 0.05 one way ANOVA, post hoc Bonferroni test). Please note that insulin and leptin concentrations for lean and sedentary and exercised obese animals have already been published (Fernandez‐Twinn et al. 2017).

### Maternal tissue insulin/MAPK/mTORC1 signaling

**Figure 1:**
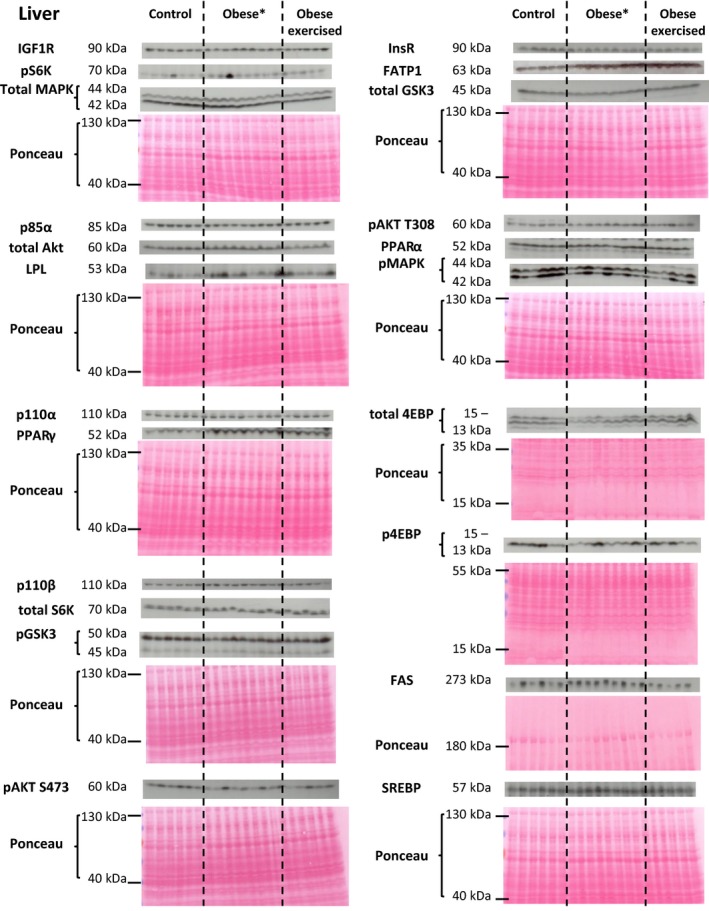
Western blots showing the abundance of insulin/MAPK/mTORC1 signaling and lipid metabolic proteins in the maternal liver. *Although nine obese sedentary dams were originally used when analysing protein expression in the maternal liver, two dams (the 4th and 5th obese liver samples) were later excluded as they did not fulfil the criteria required for inclusion in the study (as described in the Materials and Methods). The molecular weights of detect proteins are shown. The ponceau‐stained membrane for each detected protein (or series of detected proteins) shows loading for each sample and is displayed beneath the scanned western blots.

**Figure 2:**
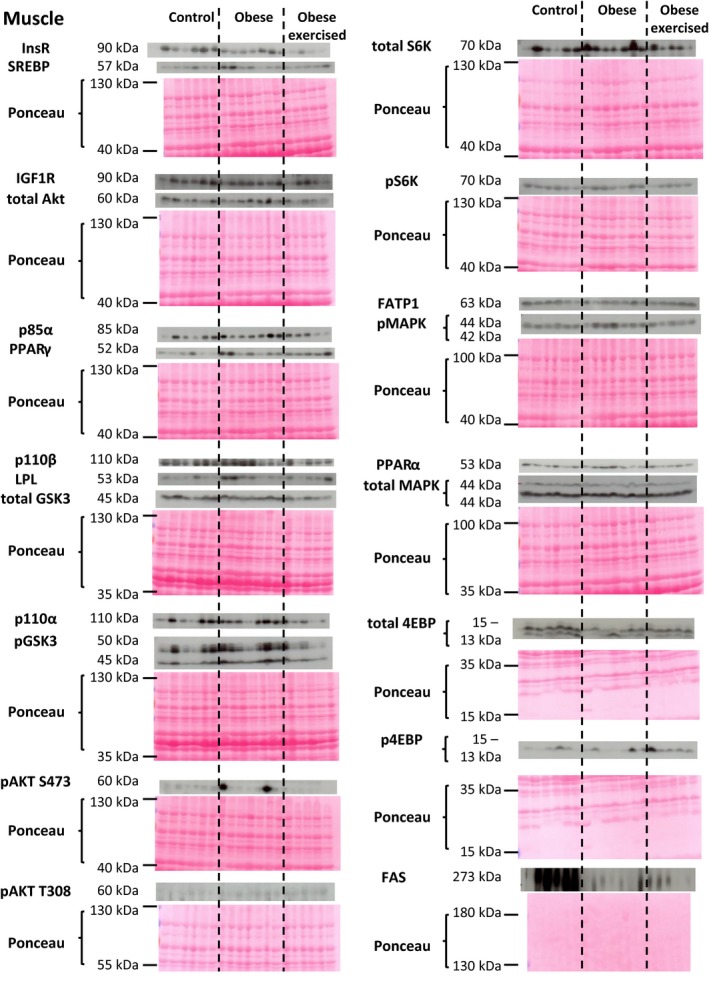
Western blots showing the abundance of insulin/MAPK/mTORC1 signaling and lipid metabolic proteins in the maternal skeletal muscle. The molecular weights of detect proteins are shown. The ponceau‐stained membrane for each detected protein (or series of detected proteins) shows loading for each sample and is displayed beneath the scanned western blots.

**Figure 3:**
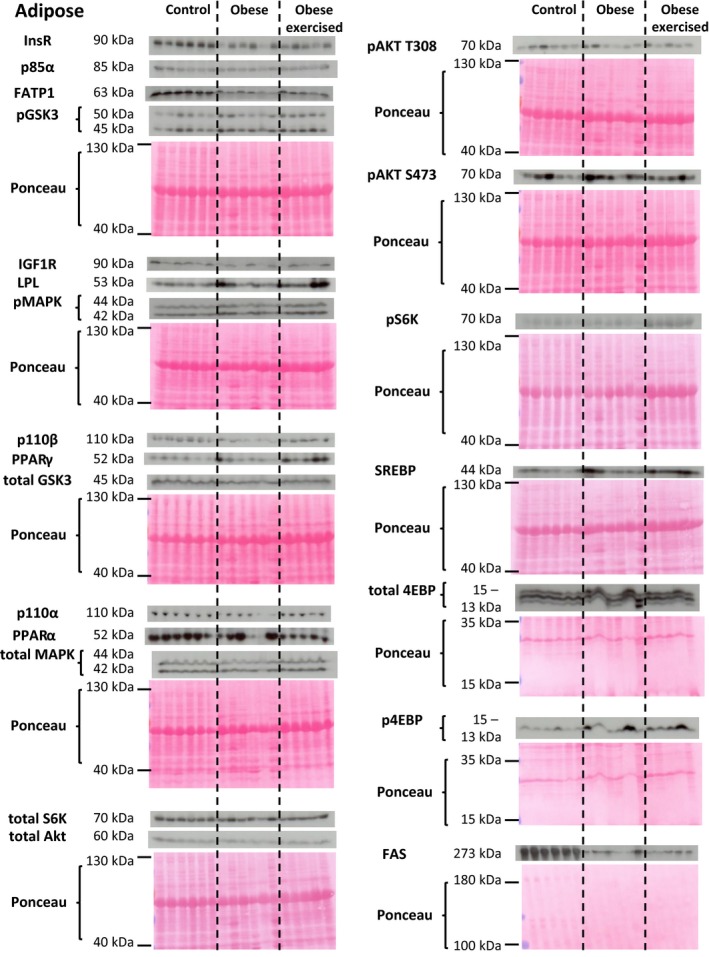
Western blots showing the abundance of insulin/MAPK/mTORC1 signaling and lipid metabolic proteins in the maternal white adipose tissue. The molecular weights of detect proteins are shown. The ponceau‐stained membrane for each detected protein (or series of detected proteins) shows loading for each sample and is displayed beneath the scanned western blots.

**Figure 4:**
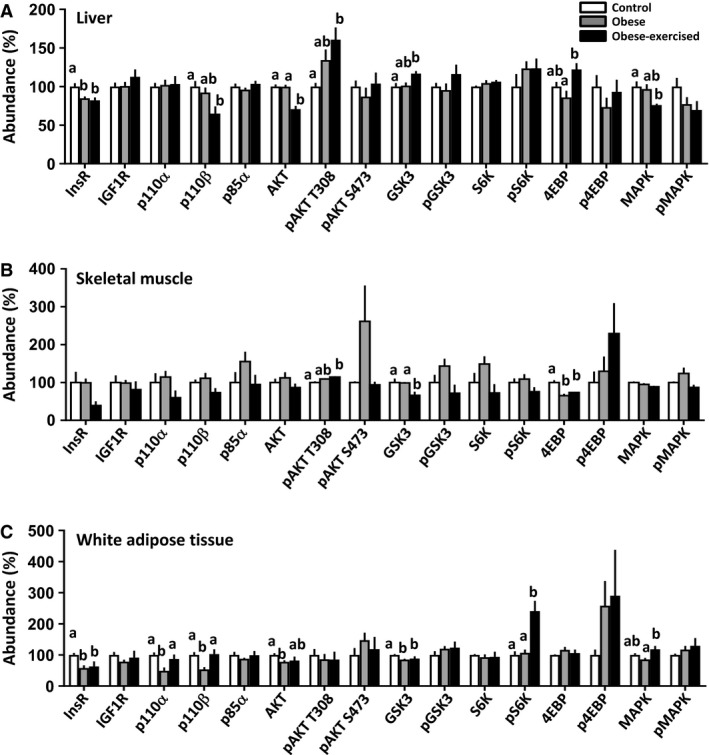
Abundance of insulin/MAPK/mTORC1 signaling proteins in the maternal liver (A), skeletal muscle (B) and white adipose tissue (C). Data are mean ± SEM percentage of the control group from control (*n* = 6), obese (*n* = 6–7) and obese‐exercised (*n* = 5). Values with different superscript letters are significantly different from each other (*P* < 0.05, one‐way ANOVA with Bonferroni post hoc).

The abundance of metabolic proteins in the insulin/MAPK/mTORC1 signaling pathway in the maternal liver, skeletal muscle and white adipose tissue was assessed in lean and sedentary and exercised obese dams (western blots are shown in Figs. 1, 2, 3 and quantification of protein abundance is shown in Fig. 4).

#### Liver

Exercise had a more marked effect on the insulin/MAPK/mTORC1 signaling pathway than obesity per se (Figs. 1 and 4A). Only the insulin receptor was altered in the sedentary obese dams with reduced abundance compared to the control dams (Figs. 1 and 4A). This change remained in the exercised obese dams, but were now also accompanied by reduced abundance of p110*β*, AKT, and MAPK and increased levels GSK3 and 4EBP relative to either the sedentary obese dams or to both the sedentary and control groups (Figs. 1 and 4A).

#### Skeletal muscle

Insulin/MAPK/mTORC1 signaling in the skeletal muscle was largely unaffected by obesity alone but was influenced more extensively by exercising the obese dams (Figs. 2 and 4B). Only 4EBP abundance was significantly altered in skeletal muscle of sedentary obese relative to the control dams, with maternal obesity causing a decrease in 4EBP (Figs. 2 and 4B). Exercising the obese dams produced more changes in this signalling pathway, with an increased abundance of pAKT T308 a paradoxical decreased abundance of GSK compared to skeletal muscle of the obese sedentary and/or control groups (Figs. 2 and 4B). In contrast to the liver, skeletal muscle MAPK signaling proteins were not affected by exercising obese dams (Figs. 2 and 4B).

#### White adipose tissue

Obesity reduced WAT insulin/MAPK/mTORC1 signaling, which was partially reversed by exercising the obese dams (Figs. 3 and 4C). Obesity decreased WAT abundance of InsR, the PI3K catalytic subunits (p110*α* and p110*β*), total Akt, and total GSK3 relative to the control group (Figs. 3 and 4C). Exercise restored p110*α* and p110*β* abundance to control values and increased expression of pS6K and total MAPK relative to values in control dams and/or sedentary obese dams (Figs. 3 and 4C).

### Maternal tissue lipid metabolism

**Figure 5:**
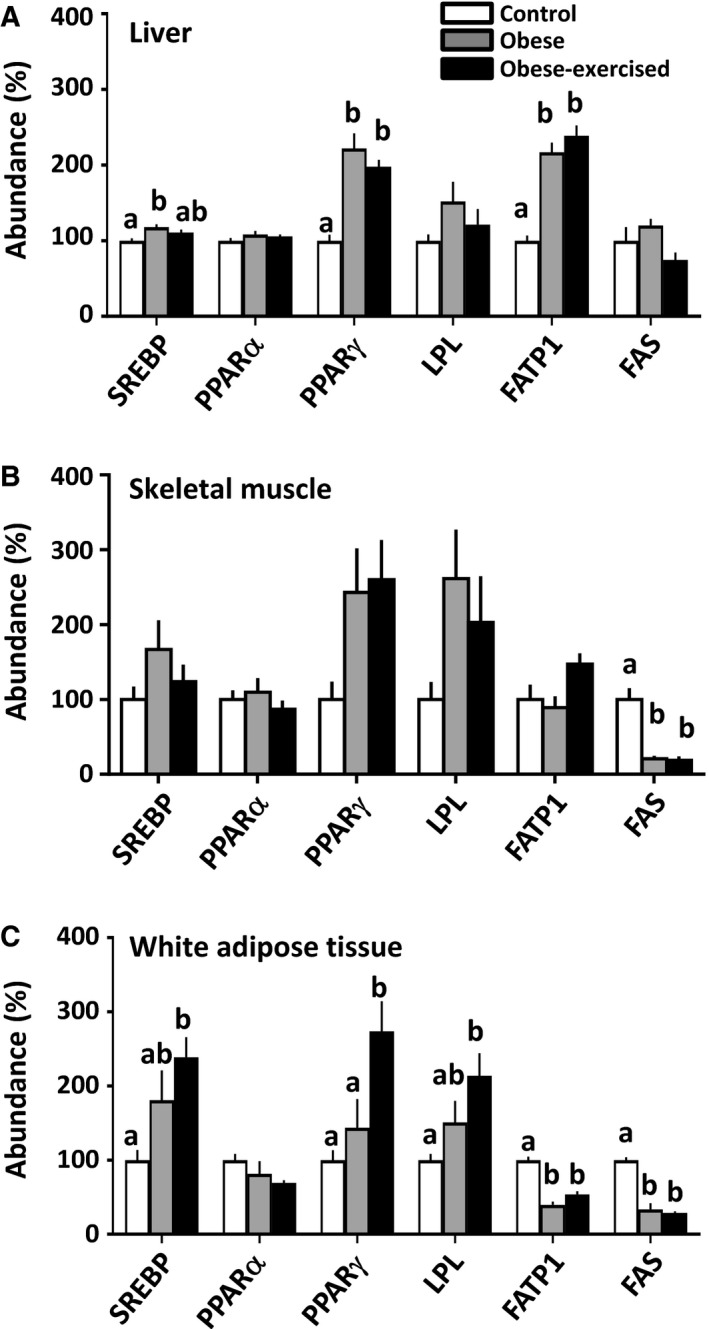
Abundance of lipid metabolic proteins in the maternal liver (A), skeletal muscle (B) and white adipose tissue (C). Data are mean ± SEM percentage of the control group from control (*n* = 6), obese (*n* = 6–7) and obese‐exercised (*n* = 5). Values with different superscript letters are significantly different from each other (*P* < 0.05, one‐way ANOVA with Bonferroni post hoc).

The abundance of proteins involved in lipid metabolism in the maternal liver, skeletal muscle, and white adipose tissue was assessed in lean and sedentary and exercised obese dams (western blots are shown in Figures 1, 2, 3 and quantification of protein abundance is shown in Fig. 5).

#### Liver

Hepatic abundance of SREBP, PPAR*γ*, and FATP1 was increased in sedentary obese dams (Figs. 1 and 5A). Both PPAR*γ*, and FATP1 abundance remained elevated in the obese exercised dams, whereas SREBP was no longer significantly difference from control values (Figs. 1 and 5A). Neither PPAR*α*, FAS, nor LPL were affected in the sedentary and exercised obese dams relative to the control group (Figs. 1 and 5A).

#### Skeletal muscle

Obesity had little effect on the proteins of lipid metabolism in skeletal muscle (Figs. 2 and 5B). Abundance of FAS alone was significantly lower in both obese groups than in the controls (Figs. 2 and 5B). All other lipid metabolic proteins assessed were unaffected by obese, regardless if they were sedentary or exercised relative to the control group (Figs. 2 and 5B).

#### WAT

Exercise had little effect on the WAT abundance of FATP1 and FAS, which was reduced to a similar extent in the two obese groups relative to control values (Figs. 3 and 5C). Exercising obese dams increased WAT abundance of both SREBP and LPL compared to control values with intermediate values in the sedentary obese dams (Fig. 3 and 5C). Exercise also elevated PPAR*γ* expression in the WAT of obese dams relative to the other two groups (Figs. 3 and 5C).

### Relationships between maternal metabolic parameters

**Figure 6:**
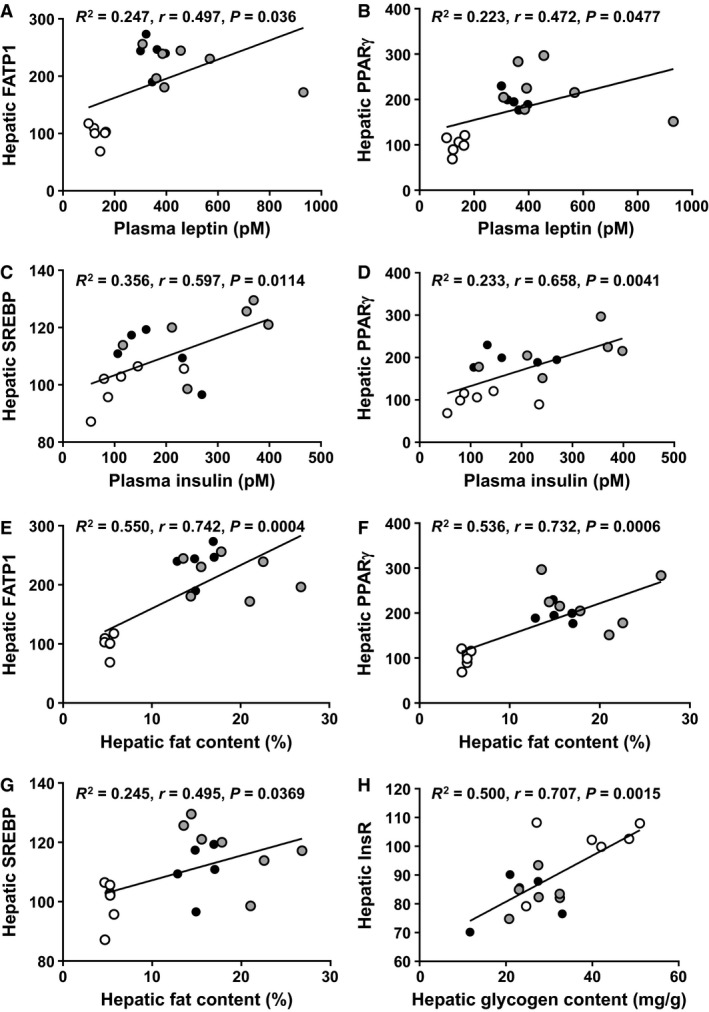
The relationship between lipogenic and insulin signaling proteins in the liver with maternal plasma leptin and insulin and tissue fat and glycogen content. Control *n* = 6, white circle, obese *n* = 7, gray circle and obese‐exercised *n* = 5, black circle). The results of the correlations are show in Table 4.

We then investigated if maternal hormone concentrations and biochemical composition were associated with the abundance of metabolic proteins in the maternal tissues studied at the end of pregnancy by combining the data from all three groups of dams (Fig. 6 and Table 5). Circulating leptin correlated positively with hepatic FATP1 and PPAR*γ*, while insulin concentrations correlated positively with hepatic SREBP and PPAR*γ* (Fig. 6A–D). In contrast, circulating insulin correlated negatively p110*α* and FATP1 abundance in the WAT of the dam (Table 5). Maternal liver fat content correlated positively with FATP1, SREBP and PPAR*γ* (Fig. 6E–G). Hepatic glycogen content‐associated positively with InsR and MAPK, but correlated negatively with total GSK3 (Fig. 6H and Table 5). Maternal triglyceride concentrations correlated positively to FAS in the skeletal muscle and to FAS and FATP1 in the WAT (Table 5). Maternal glucose tolerance, which has been reported previously (Fernandez‐Twinn et al. 2017), was correlated positively with hepatic PPAR*γ* and fat content, but negatively correlated with white adipose tissue FATP1 and FAS (Table 5).

**Table 5:** Relationships between maternal metabolic parameters.

Parameters	Coefficient of determination (*R* ^2^ value)	Correlation coefficient (*r* value)	*P* value
Leptin versus hepatic FATP1	0.247	0.497	0.0360
Leptin versus hepatic PPAR*γ*	0.223	0.472	0.0477
Insulin versus hepatic SREBP	0.356	0.597	0.0114
Insulin versus hepatic PPAR*γ*	0.433	0.658	0.0041
Hepatic fat content versus hepatic FATP1	0.550	0.742	0.0004
Hepatic fat content versus hepatic PPAR*γ*	0.536	0.732	0.0006
Hepatic fat content versus hepatic SREBP	0.245	0.495	0.0369
Hepatic glycogen content versus hepatic InsR	0.500	0.707	0.0015
Hepatic glycogen content versus hepatic MAPK	0.275	0.524	0.0308
Hepatic glycogen content versus hepatic GSK3	0.252	−0.502	0.0401
Triglycerides versus skeletal muscle FAS	0.565	0.752	0.0003
Triglycerides versus WAT FATP1	0.393	0.627	0.0071
Triglycerides versus WAT FAS	0.601	0.775	0.0003
Insulin versus WAT FATP1	0.397	−0.630	0.0089
Insulin versus WAT p110*α*	0.353	−0.594	0.0152
AUC GTT versus hepatic PPAR*γ*	0.436	0.661	0.0039
AUC GTT versus hepatic fat content	0.335	0.579	0.0149
AUC GTT versus WAT FATP1	0.356	−0.596	0.0147
AUC GTT versus WAT FAS	0.349	−0.591	0.0160

The relationships between parameters were measured in all groups combined (control *n* = 5–6, obese *n* = 6–7, obese‐exercised *n* = 5) using Pearson’s correlations. Please note that AUC GTT and insulin and leptin concentrations for lean and sedentary and exercised obese animals have already been published (Fernandez‐Twinn et al. 2017). AUC GTT = area under the curve for glucose tolerance test.

## Discussion

This study shows that a short period of daily exercise from before pregnancy until close to term has beneficial effects in preventing some of the changes in tissue insulin signalling and lipid metabolic pathways seen in obese mouse dams. Exercise also induced changes in specific proteins of the insulin signalling and lipid metabolic pathways in obese dams that were different to control and obese sedentary dams. The molecular changes induced by obesity alone and in response to exercising the obese dams were tissue specific and, in some instances, were related to alterations in maternal biochemical composition and concentrations of specific hormones and metabolites in late pregnancy. There were no beneficial effects of maternal exercise on the fetal growth restriction induced by maternal obesity despite the long term beneficial effects of the current exercise intervention in obese dams on cardiac (Beeson et al. 2018) and metabolic health (Fernandez‐Twinn et al. 2017) in adult offspring. Importantly this suggests that it is not fetal growth restriction *per se* that may mediate the long‐term detrimental health consequences of maternal obesity on the offspring (Fernandez‐Twinn et al. 2017; Beeson et al. 2018). Taken together, our data highlight the specific physiological and molecular mechanisms operating during pregnancy that mediate the beneficial effects of exercise on maternal and offspring outcomes in obese pregnancies. These findings are relevant for identifying potential metabolic targets for therapeutic intervention and reinforce the benefit of lifestyle strategies in reducing the burden of the current obesity epidemic on health care systems worldwide.

In line with the observed increased overall adiposity in both obese sedentary and exercised mice (Fernandez‐Twinn et al. 2017; Beeson et al. 2018), WAT mass expansion and fat deposition in liver, skeletal muscle and WAT was not significantly different in these two obese groups. This observation was also consistent with the maternal hyperleptinemia of both obese groups during pregnancy shown previously (Fernandez‐Twinn et al. 2017). There was also an increased abundance of lipogenic proteins including SREBP and PPAR*γ*, particularly in the liver, of both sedentary and exercised obese dams. The abundance of lipogenic proteins and fat content in the maternal liver was correlated positively with maternal plasma insulin and a reduced clearance of glucose upon challenge, which may suggest a causative role for hyperinsulinemia and glucose intolerance in stimulating fat accumulation in insulin‐sensitive tissues of obese dams. Plasma triglyceride concentrations were lower in obese dams, regardless of whether they were sedentary or exercised. This was possibly secondary to an enhanced storage of triglyceride in maternal tissues. However, the abundance of other lipogenic proteins (FAS in the skeletal muscle and FAS and FATP1 in the WAT) were decreased in sedentary and exercised obese dams, which may have minimised further lipid deposition in those tissues.

The obesity‐induced hypotriglyceridemia and changes in hepatic PPAR*γ*, skeletal muscle FAS, and white adipose tissue FATP1 and FAS are similar to that observed for pregnant rodents fed an obesogenic diet during pregnancy alone (Jen et al. 1991; Musial et al. 2017). In the obese dams of this study, the exercise intervention upregulated PPAR*γ* in the WAT. The intervention also accentuated the obesity‐induced induction of SREBP and LPL in the maternal WAT, compared to the lean control group. There are similar alterations in the abundance of lipid metabolic proteins in skeletal muscle, liver and WAT of nonpregnant obese animals in response to exercise that are associated with the specific lipid profiles of those tissues (Rector et al. 2008; Ritchie et al. 2011; Higa et al. 2014; Jordy et al. 2015). Thus, although there was no difference in total tissue fat content between sedentary and exercised obese dams, there may be differences in tissue uptake, synthesis, and oxidation of specific lipids that could be analysed in further studies.

Like tissue lipid content, the exercise intervention did not alter the protein tissue content in obese dams. In particular, tissue protein content of the liver and WAT were similarly reduced in both obese sedentary and exercised dams. However, hepatic glycogen content was reduced specifically in the obese exercised dams compared to the lean control group. The abundance of GSK3 and mTORC1 signaling, which negatively regulates glycogen synthesis and promotes protein synthesis, respectively, were altered in the maternal liver and WAT, as well as in the skeletal muscle by exercising the obese dams. These molecular changes may have been important for the re‐synthesis of glycogen, translation of myofibrillar proteins and potential synthesis of new adipocytes that are insulin sensitive, in response to the 20 min daily exercise intervention in the obese dams (Jensen and Richter 2012; Egan and Zierath 2013; Long et al. 2014).

Similar to tissue fat content, exercise did not ameliorate the obesity‐induced decrease in the InsR and accentuated the obesity‐induced upregulation of AKT activation (pAKT T308) in the maternal liver, compared to lean controls. Genetic loss of AKT is known to prevent hepatic accumulation of lipids in response to high dietary fat consumption (Leavens et al. 2009). Thus, the postreceptor activation of AKT may have been responsible for the fatty liver in obese exercised dams, seen here. However, hepatic abundance of phosphoinositol 3‐kinase (PI3K)‐p110*β*, total AKT and total MAPK were diminished by exercise in obese dams, in line with the reduction in maternal insulin concentrations reported previously in exercised relative to sedentary obese dams (Fernandez‐Twinn et al. 2017). Mouse dams fed an obesogenic diet only during pregnancy also displayed increased hepatic insulin sensitivity (eg, increased AKT activation), which was associated with diminished glucose production by the liver (Musial et al. 2017). However, further work is required to determine whether endogenous glucose production in the obese dams of this study whose obesity preexisted prepregnancy, is also reduced and importantly, rescued by exercise‐induced changes in hepatic insulin sensitivity.

Skeletal muscle insulin signaling was the least affected by obesity but was altered by exercising obese dams. In particular, the abundance of GSK3 was decreased in obese dams in response to the exercise intervention. Although, there was an upregulation of AKT activation (pAKT T308) in the skeletal muscle of exercised obese dams compared to lean controls. This is in contrast to findings in nonpregnant animals, where exercise instead upregulates the abundance of several insulin signalling components in the skeletal muscle (Goodyear et al. 1996; Hayashi et al. 1997; Kim et al. 1999; Chibalin et al. 2000; Hawley and Lessard 2008). Unlike the maternal skeletal muscle, the abundance of several components of the insulin signaling pathway was downregulated in the WAT of the obese dam (insulin receptor, PI3K‐p110*α*, PI3K‐p110*β*, AKT, and GSK3). Moreover, a few components of the insulin signaling pathway were restored to control levels by exercising the obese dams (PI3K‐p110*α* and PI3K‐p110*β*). We have previously found that the exercise intervention improved maternal insulin sensitivity and glucose intolerance of obese dams (Fernandez‐Twinn et al. 2017). Moreover, the acquisition of insulin resistance that accompanies normal pregnancy is linked to changes insulin signalling components in the skeletal muscle and WAT (Saad et al. 1997; Barbour et al. 2007; Musial et al. 2016). Thus, it is highly likely that there are interactions between obesity, exercise, and pregnancy in determining the profile of protein expression in each maternal tissue. However, taken together, our data provide important evidence that insulin resistance of the mother’s WAT may be the cause of poor glucose‐insulin handling in obese pregnancies, which is modifiable by maternal exercise. In support of this notion, maternal WAT abundance of PI3K‐p110*α*, FATP1 and FAS correlated negatively with maternal insulin concentration and/or maternal glucose tolerance (Fernandez‐Twinn et al. 2017). Furthermore, previous work also reported improved whole body glucose disposal and insulin signaling in the WAT, but not skeletal muscle, of obese mice with voluntary access to a running wheel during pregnancy (Carter et al. 2015). Several of the parameters measured were not affected by exercising the obese dams. This may relate to the variability observed between obese dams (for both sedentary and exercised). It may also suggest that other molecular signaling pathways such as those involved in energy sensing (AMPK) or additional physiological systems like the cardiovascular system, which were not assessed, may play roles in mediating the beneficial effects on exercise on the pregnancy outcome of obese dams. Although only a handful of changes induced by obesity were corrected by exercise in obese dams, the long‐term effects of the exercise intervention on the metabolic health of obese mothers postpartum, require elucidation. Moreover, the effect of exercise on maternal tissue insulin signalling and whole body glucose and insulin handling in lean mothers during pregnancy should be analysed in future work. Finally, further work should also compare the effect of exercise and obesity on the metabolic profile of nonpregnant and pregnant animals. This would help to identify the contribution and interaction of obesity, exercise, and the pregnant state to the metabolic phenotype of the animal.

In common with previous findings in rats (Vega et al. 2015) and mice (Stanford et al. 2015a; Beeson et al. 2018), maternal exercise was not able to prevent the obesity‐induced reduction in fetal/offspring birth weight. However, despite the lack of effect on fetal growth, we have shown previously that the exercise intervention used in this study prevented the development of placental hypoxia and lipidemia, as well as male offspring insulin resistance, cardiac hypertrophy, and cardiac dysfunction due to maternal obesity (Fernandez‐Twinn et al. 2017; Beeson et al. 2018). Similar beneficial effects of exercise on the cardiovascular and metabolic health of the offspring have been reported in overweight or obese women (Barakat et al. 2016; Patel et al. 2017) and in other experimental animals (Carter et al. 2012; Carter et al. 2013; Rajia et al. 2013; Laker et al. 2014; Blaize et al. 2015; Raipuria et al. 2015; Vega et al. 2015; Stanford et al. 2015a; Quiclet et al. 2017; Ribeiro et al. 2017; Stanford et al. 2017; Cunningham et al. 2018). Our previous work has shown that maternal hyperinsulinemia is a predictor of offspring cardiometabolic health in obese pregnancies (Fernandez‐Twinn et al. 2017). Moreover, metabolic changes in the organs of the obese mother (with and without exercise) can impact whole body insulin sensitivity and thus, whole body insulin levels. Although insulin may not cross placenta, it can affect placental functions, such as nutrient supply to the fetus (Sferruzzi‐Perri et al. 2013a; Fowden et al. 2014), which in turn, determines fetal growth and development. Further work is, however, required to uncover how metabolic changes in the mother with obesity (both sedentary and exercised) are transmitted to the developing organs in the fetus and their relevance for long‐term offspring cardiometabolic health.

In conclusion, our data demonstrate that insulin sensitivity of the maternal white adipose is a key factor mediating the reduced glucose tolerance of the mother due to diet‐induced obesity. Furthermore, these data indicate that restoring insulin sensitivity of obese mothers during pregnancy using an exercise intervention (Fernandez‐Twinn et al. 2017) is at least partly due to beneficial changes in WAT insulin sensitivity. These findings are relevant for identifying potential sites of metabolic targets to prevent the development of insulin resistance and metabolic syndrome. They also reinforce the benefit of lifestyle strategies in reducing the burden on health care systems worldwide of the current obesity epidemic.

## Conflict of Interest

The authors have no conflicts of interest to declare.
